# Inter-observer Agreement and Reproducibility of Pertrochanteric Fracture Classification Using Plain Radiograph Versus Computed Tomogram Images: A Study of 523 Patients

**DOI:** 10.7759/cureus.48413

**Published:** 2023-11-06

**Authors:** Mitsuaki Noda, Shunsuke Takahara, Atsuyuki Inui, Shin Osawa, Takehiko Matsushita

**Affiliations:** 1 Orthopaedics, Nishi Hospital, Kobe, JPN; 2 Department of Orthopaedics, Hyogo Prefectural Kakogawa Medical Center, Kakogawa, JPN; 3 Department of Orthopaedic Surgery, Kobe University Graduate School of Medicine, Kobe, JPN; 4 Department of Orthopedics, Himeji Saint Mary's Hospital, Himeji, JPN; 5 Department of Orthopaedics, Kobe University Graduate School of Medicine, Kobe, JPN

**Keywords:** fracture classification, inter-observer agreement, computed tomogram, plain radiograph, pertrochanteric fracture

## Abstract

Background

A precise preoperative imaging classification system for pertrochanteric fractures is imperative due to the reported unreliability of the current classification system, which relies solely on plain radiographs. This study aims to achieve two primary objectives: (i) elucidate the reproducibility of pertrochanteric fracture evaluation based on the Revised Arbeitsgemeinschaft für Osteosynthesefragen/Orthopedic Trauma Association (AO/OTA) Classification, comparing plain radiographs and computed tomography (CT) scan images, and (ii) investigate the consistency of fracture classification between both imaging modalities.

Methods

A total of 523 patients (112 males and 411 females, mean age 85 years) who had both preoperative plain radiographic and three-dimensional CT images were enrolled in this study. Following the Revised AO/OTA Classification, three individual observers initially classified the fractures in plain radiograph images as either Stable (A1) or Unstable (A2). Subsequently, they further categorized them into five sub-categories (A1.1, A1.2, A1.3, A2.2, and A2.3). The same classification system was applied to the CT scan images. Inter-observer agreement and consistency of fracture classification between plain radiographs and CT scan images were assessed.

Results

The inter-observer agreement for fractures classified as stable or unstable using only plain radiographs was found to be fair among the three observers, with a mean κ of 0.397 (95% CI: 0.316-0.478). However, inter-observer agreement improved significantly when using CT scans, with a mean κ of 0.590 (95% CI: 0.518-0.662). Our results demonstrated a consistency level between two graphical modalities ranging from fair to moderate, with κ values of 0.581, 0.383, and 0.335, respectively. It's worth noting that plain radiographic classification occasionally resulted in underestimations, with each observer identifying 16.1%, 34.0%, and 37.9%, respectively, of cases as A1 in plain radiographs that were classified as A2 in CT scans.

Conclusions

This study reveals a moderate to substantial level of inter-observer agreement for fracture classification when using CT scan images, in contrast to plain radiographs. Fracture evaluation relying solely on plain radiographs sometimes underestimates fracture classification and exhibits less consistency compared to using CT scan images.

## Introduction

The incidence of femoral pertrochanteric fractures among elderly patients is steadily rising on a global scale [1]. Consequently, it becomes imperative for surgeons to bestow due diligence upon the application of a precise preoperative imaging classification system, as it profoundly impacts postoperative outcomes. The precision in classifying these fractures is paramount, first, influencing the selection of optimal implants, such as dynamic hip screws (DHS) for stable pertrochanteric fractures and intra-medullary nails (IMN), a modified DHS for unstable fractures, or hemiarthroplasty [2-4]. In cases where stability is elusive, femoral locking compression plates, rather than DHS, have been advocated as an alternative device [5]. The significance of the correct implant choice is underscored by a meta-analysis revealing superior Parker scores with IM nails in unstable trochanteric fractures as opposed to DHS [6]. Second, clinical and epidemiological studies comparing stable and unstable pertrochanteric fractures may suffer a loss of credibility when misclassified [7-9].

In clinical practice, the ideal fracture classification system should be concise, encompass stability assessment, and most crucially, exhibit a notable degree of reproducibility for widespread adoption. Among the multitude of pertrochanteric fracture classification systems, the original Arbeitsgemeinschaft für Osteosynthesefragen (AO Foundation)/Orthopedic Trauma Association (AO/OTA) Classification enjoyed wide usage [10]. Despite its relatively weak inter-observer agreement, it outperformed the Evans, Kyle, and Boyd classification systems [11,12]. On the other hand, the Revised 2018 AO/OTA Classification [10], a simplified version of the original, has demonstrated enhanced reproducibility compared to the aforementioned classification systems [13]. Nevertheless, this revised type still presents moderate to low inter-observer reliability when relying solely on plain radiograph images [14,15]. This decrease in reproducibility can be attributed to the inherent challenges in interpreting fractures from antero-posterior (AP) and lateral plain radiograph images.

Advanced imaging modalities, particularly computed tomography (CT) scans, offer a promising avenue for improving the reproducibility of pertrochanteric fracture evaluation using the Revised AO/OTA Classification, much as they have with the Original AO Classification [16]. CT scan images provide an in-depth view of the proximal femur's morphology, particularly in the posterolateral and posteromedial regions [13,14]. Notably, an evaluation of pertrochanteric fractures using the Revised AO/OTA Classification, with the aid of 3-dimensional CT scan images, is yet to be undertaken on a large scale. Additionally, a comparative analysis of fracture classification between plain radiographs and CT scan images remains uncharted territory. This study seeks to accomplish two objectives: (i) to elucidate the reproducibility of inter-observer agreement in pertrochanteric fracture classification, comparing plain radiographs with CT scan images based on the Revised AO/OTA Classification, and (ii) to investigate the consistency of fracture classification interpretation between plain radiographs and CT scan images. We hypothesize that the utilization of CT scan images will enhance inter-observer agreement compared with the assessment based solely on plain radiographs. Furthermore, we anticipate that plain radiographic evaluation will exhibit a weak correlation when contrasted with CT scan evaluation.

Ethics

This study garnered approval from the Research Ethics Board of all participating hospitals, (represented by the Nishi hospital Institutional Review Board (IRB) committee with approval number of 2021-1).

## Materials and methods

Data source and collection

Data was gathered retrospectively from orthopedic patients across four hospitals. We identified a cohort of 635 patients with pertrochanteric fractures from the Surgical Entry Database of these hospitals, spanning from January 2015 to December 2020. The study included patients with preoperative two-directional plain radiographs (anteroposterior and lateral views) and preoperative three-dimensional (3D) CT scans. Exclusion criteria were meticulously applied, encompassing the following: (1) AO/OTA A3 Type fractures, which were categorized as subtrochanteric fractures in some hospital records, leading us to omit the A3 type to ensure data uniformity; (2) pathologic fractures and/or the presence of osteoarthritic changes; (3) prior history of ipsilateral hip fracture; and (4) age below 30 years at the time of the injury.

**Figure 1:**
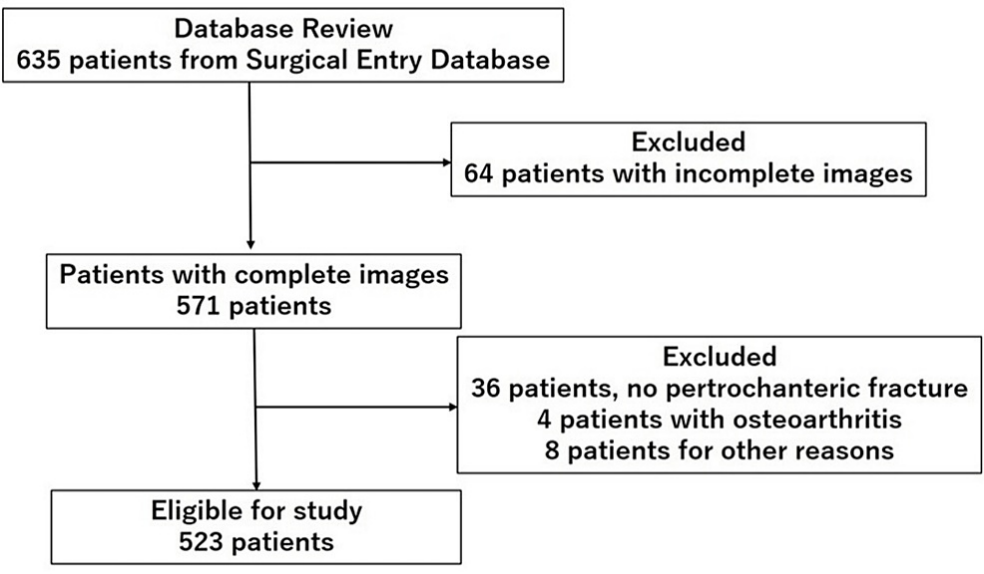
CONSORT flow diagram showing patient inclusion and exclusion CONSORT: Consolidated standards of reporting trials

Out of the initially identified 635 patients from the Surgical Entry Database, a total of 523 patients were deemed eligible for the study. Among these, 112 patients were male, and 411 were female, with a mean age at the time of surgery being 85 years (ranging from 38 to 103 years). The laterality of the fractures was distributed as 298 on the left side and 225 on the right side. Sixty-four patients due to an incomplete set of images (52 patients for CT and 12 patients for plain radiographs), the absence of pertrochanteric fractures (36 patients), moderate osteoarthritic changes (four patients), and various other reasons (eight patients) were excluded from the current study (Figure 1).

Data classification

The classification of pertrochanteric fractures was based on the Revised AO/OTA Classification. Initially, fractures were categorized as either stable (A1) or unstable (A2) and subsequently subcategorized into five distinct subgroups (A1.1, 1.2, 1.3, A2.2, and A2.3) [16].

Three orthopedic surgeons were enlisted as observers, comprising one orthopedic chief resident and two senior surgeons, each with a professional tenure of 20 years or more. These observers routinely reviewed proximal femoral radiographs in their daily practice and possessed a comprehensive understanding of both the Original and the Revised AO/OTA Classifications prior to the commencement of this study.

The observers initiated the classification process for each case exclusively utilizing plain radiographs (anteroposterior (AP) and lateral views) and subsequently repeated the classification for each case with 3D CT scan images at separate time intervals. To emulate a clinical setting, each observer was allotted ample time for assessment, with strict prohibitions on discussing findings with their peers.

Statistical analysis

Inter-observer agreement was meticulously assessed among the three observers, involving the calculation of Kappa coefficient values, along with their corresponding 95% confidence intervals. Moreover, the concordance in fracture classification using plain radiographs versus CT scan images was examined individually for each of the three observers.

For the statistical analysis of observer reproducibility, we employed EZR software (Saitama Medical Center, Jichi Medical University, Saitama, Japan) a graphical interface for R software (The R Foundation for Statistical Computing, Vienna, Austria). The Kappa coefficient (κ) was interpreted as follows: poor agreement (less than 0.00); slight agreement (0.00-0.20); fair agreement (0.21-0.40); moderate agreement (0.41-0.60); substantial agreement (0.61-0.80); and almost perfect agreement (0.81-1.00) [17]. Additionally, the confidence intervals (CI) for the three observers, based on a sample size of 60 patients, were calculated, with a lower limit of 0.5 and an upper limit of 0.8 (α = 0.05) [16].

## Results

Inter-observer agreement

In the context of simplified fracture classification into stable (A1) or unstable (A2) categories, the inter-observer agreement based on plain radiographs was represented by Kappa values within 95% Confidence Intervals, yielding values of 0.455 (0.377- 0.532), 0.379 (0.296- 0.463), and 0.358 (0.276- 0.440). The average agreement was characterized as "fair," with κ = 0.397 (0.316 - 0.478). In contrast, the inter-observer agreement based on CT scan images was reflected in Kappa values and their 95% Confidence Intervals, resulting in values of 0.531 (0.456- 0.606), 0.741 (0.675- 0.806), and 0.498 (0.422- 0.575), with the mean agreement elevating to "substantial," resulting in κ = 0.590 (95% CI: 0.518 - 0.662) (Table 1).

**Table 1:** Inter-observer agreement in the simplified classification of pertrochanteric fractures as stable (A1) or unstable (A2) Kappa coefficient and 95% confidence interval values, in parenthesis, with their means are shown. Observers 1 and 2 are orthopedic trauma surgeons and Observer 3 is an orthopedic resident.

Observer	Plain radiographs	CT
1vs2	0.455 (0.377~0.532)	0.531 (0.456~0.606)
2vs3	0.379 (0.296~0.463)	0.741 (0.675~0.806)
1vs3	0.358 (0.276~0.440)	0.498 (0.422~0.575)
Mean	0.397 (0.316~0.478)	0.590 (0.518~0.662)

For the complete classification involving five groups, the inter-observer agreement with plain radiographs was represented by Kappa values and their 95% Confidence Intervals, yielding values of 0.340 (0.279-0.402), 0.422 (0.356-0.486), and 0.262 (0.201-0.323). The overall mean agreement based on plain radiographs was described as "fair," with κ = 0.341 (95% CI: 0.279 - 0.404). In contrast, CT scanning exhibited agreement values of 0.460 (0.399-0.522), 0.705 (0.652-0.759), and 0.479 (0.419-0.539), with the mean agreement termed as "moderate," κ= 0.548(95% CI: 0.490 - 0.607) (Table 2).

**Table 2:** Inter-observer agreement in the complete classification of pertrochanteric fractures (A1.1, A1.2, A1.3, A2.2, A2.3) Kappa coefficient and 95% confidence interval values, in parenthesis, with their means are shown. Observers 1 and 2 are orthopedic trauma surgeons and Observer 3 is an orthopedic resident.

Observer	Plain radiographs	CT
1vs2	0.340 (0.279~0.402)	0.460 (0.399~0.522)
2vs3	0.422 (0.356~0.486)	0.705 (0.652~0.759)
1vs3	0.262 (0.201~0.323)	0.479 (0.419~0.539)
Mean	0.341 (0.279~0.404)	0.548 (0.490~0.607)

Consistency of fracture classification using plain radiograph vs. CT scan images

Our results indicated a "fair" to "moderate" agreement in the simplified fracture classification (two groups) between plain radiographic and CT scan images, with values of κ = 0.581 (95% CI: 0.511 - 0.650), 0.383 (95% CI: 0.309 - 0.456), and 0.335 (95% CI: 0.262 - 0.408) among the three observers, respectively. The percentage of exact estimations for plain radiographic and CT scan classification was 78.8%, 66.0%, and 62.1% for the three observers, respectively. Instances of underestimation in plain radiographic classification, such as categorizing A1 in plain radiographs as A2 in CT scans, were observed in 16.1%, 34.0%, and 37.9% of cases for the three observers, respectively. Overestimations accounted for 5.2%, 0%, and 0%, respectively (Table 3).

**Table 3:** Consistency of pertrochanteric fracture classification as stable (A1) or unstable (A2) between the three observers using plain radiographs versus CT scan images ‡ Kappa coefficient and 95% confidence interval values, in parenthesis, with their means are shown. Observers 1 and 2 are orthopedic trauma surgeons and Observer 3 is an orthopedic resident.

Observer	Under-estimate (%)	Exact estimate (%)	Over-estimate (%)	‡Kappa coefficient
1	16.1 (84/523)	78.8 (412/523)	5.2 (27/523)	0.581 (0.511-0.650)
2	34.0 (178/523)	66.0 (345/523)	0	0.383 (0.309-0.456)
3	37.9 (198/523)	62.1 (325/523)	0	0.335 (0.262-0.408)

In the context of the complete classification (five groups), our current findings revealed a "fair" to "moderate" agreement between plain radiographic and CT scan images, with values of κ = 0.472 (95% CI: 0.411 - 0.533), 0.343 (95% CI: 0.281 - 0.406), and 0.314 (95% CI: 0.254 - 0.375) among the three observers, respectively. The percentage of exact estimations for plain radiographic and CT scan classification was 64.6%, 55.3%, and 51.2% for the three observers, respectively. Instances of underestimation in plain radiographic classification for each observer were 27.9%, 44.4%, and 48.5%, respectively, while overestimations were observed at 7.5%, 0.4%, and 0.2%, respectively.

Case presentation

**Figure 2:**
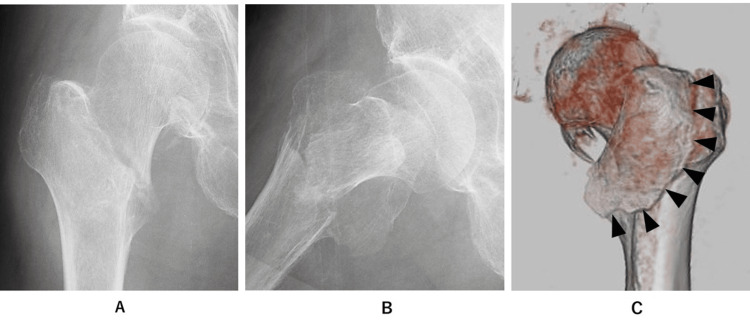
Case presentation Using two-dimensional plain radiographs (A: anterolateral view, and B: lateral view), the fracture was classified as Type A-1.3. However, CT scan images (C: 3D CT image) revealed a large-sized pertrochanteric fragment from the greater trochanter to the trochanteric crest, lesser trochanter (indicated in black arrowheads). Hence, the fracture was re-classified as Type A-2.2.

An 86-year-old male patient sustained a right-sided pertrochanteric fracture as a result of a fall. The initial classification, based on two-dimensional plain radiographs, identified the fracture as A1.3 using the Revised AO/OTA Classification. However, upon closer examination through CT scan imaging, it was reclassified as A2.2. This reclassification became necessary due to the identification of a substantial posterior fragment encompassing the posterior part of the greater trochanter, the trochanteric crest, the lesser trochanter, and the posteromedial cortex (Figure 2). It is noteworthy that relying solely on plain radiographs can occasionally lead to the oversight of sizable fractures.

## Discussion

This study has unveiled a crucial revelation - the utilization of CT scan images markedly enhances inter-observer agreement in the classification of fractures when compared to the utilization of plain radiographs. A noteworthy finding is the presence of a "fair" to "moderate" level of consistency between plain radiographic and CT scanning in the simplified classification (2 groups). This underscores a compelling observation: within the confines of plain radiographs, the detection of posterior osseous fragments is a formidable challenge. Consequently, our investigation exposes a staggering statistic, wherein 20~40% of fractures initially classified as A1 using plain radiographs were subsequently reclassified as A2 upon examination via CT scan images.

Our data on inter-observer agreement, ascertained through plain radiographs, aligns with findings in other reports [12,15]. The morphological intricacies of the posterior femoral intertrochanteric region, particularly in the sagittal plane, impede the identification of faint fracture lines in plain radiographs [2,18]. A systematic review of five published papers concluded that incorporating CT scan images into the assessment of fractures, using the original AO/OTA Classification, enhances accuracy and reproducibility, particularly in classifying pertrochanteric fractures [13]. In a separate study involving 110 patients, the application of AO/OTA classification through CT imaging exhibited robust reproducibility (κ = 0.78) [19]. It was also observed that the Original AO/OTA classification of fractures using CT scan images demonstrated substantial intra-observer reliability (κ = 0.61) and slight inter-observer reliability (κ = 0.19) [20]. However, in contrast, Cavaignac et al. [21] refuted these findings, asserting that there was no discernible improvement in fracture classification using axial sliced CT scan images (κ = 0.28). A similar report suggested that inter-observer agreement remained largely unaltered whether plain radiographs or CT scan images were employed [22].

Challenges of using plain radiographs with the revised AO/OTA classification

While the Revised AO/OTA Classification presents challenges, the integration of CT scan images serves to enhance its reliability. For instance, measuring lateral wall thickness to assess wall competence is a pivotal metric in distinguishing between A1 and A2 fractures when relying on plain radiographs [16]. Hsu CE et al. [23] defined this measurement as the distance between the fracture site and the lateral cortex. However, relying solely on plain radiographs for this measurement may introduce inaccuracies [16]. Firstly, acquiring a plain radiograph image that allows for precise measurement necessitates a traction view with the leg in a neutral rotation, a procedure that may not be easily executed in an emergency setting due to patient discomfort. Secondly, there is an absence of clinical data confirming that rotational differences in plain radiographs will not yield significant measurement errors. Lastly, recalibrating the actual distance is a time-consuming process, a step that is not required in other fracture classification systems. In our experience, 3D CT scan images provide a significantly clearer depiction of lateral wall structures in the neutral position, achieved by making simple adjustments to the rotation of the proximal femur on the monitor screen, even when dealing with a distorted position of the fractured limb. Another taxing aspect of both the Original and the Revised AO/OTA Classification is the manual counting of inter-fragmentary bone fragments, a procedure necessitated for distinguishing A2.2 from A2.3 fractures. This task can be intricate, even when utilizing CT scan images. However, it should be noted that the differentiation between A2.2 and A2.3 fractures has limited clinical significance.

Strengths and limitations

This study boasts several commendable strengths, including a substantial sample size drawn from a homogeneous cohort. Additionally, the availability of complete sets of two directional plain radiographs and 3D CT scan images for evaluation offers a robust foundation for our analysis. Furthermore, the data was drawn from multiple hospitals, encompassing a wide range of radiological images.

Nonetheless, this study does bear certain limitations. First, we did not conduct a test of intra-observer agreement, as it would have been unduly burdensome for the observers to reevaluate 523 patients. Second, the number of observers was limited to three, which is lower in comparison to other studies [13,20], and it is noteworthy that this group did not consist of an equal distribution of observers, with a mix of senior surgeons and a resident. However, it's worth noting that increasing the number of cases, rather than the number of observers, would yield a more precise reliability estimate [24]. We firmly believe that our sample size compensates for the limited number of observers in our study. Third, there may exist a bias in the selection of patients, as cases with incomplete images were excluded. Nevertheless, majority of such excluded patients didn’t seem to be purposely exempted from 3D CT, for example, limited to the stable group. Fourth, patients classified as A1.1 (greater trochanteric fracture) who underwent conservative treatment, were not included in the surgical entry database.

Future directions

Future research should focus on defining the threshold for osseous displacement and fragment size, moving beyond mere identification of bony fragments. Even when CT scan images reveal minimal bone translation, the fracture's classification as unstable, in accordance with the Revised AO/OTA Classification, should be considered. Given that such fractures retain soft tissue stability, they should be classified as stable fractures. The utilization of 3D CT reconstruction can offer a deeper understanding of the morphological characteristics of bone fragments, enabling not only their detection but also quantification [25,26]. Nonetheless, future innovations must also endeavor to explore techniques that mitigate the need for excessive CT radiation exposure. The conversion of plain radiographic images into computerized 3D images is facilitated by advanced imaging techniques. Alternatively, the development of novel approaches for the prediction of stable fractures, akin to the innovative Posterior Protrusion Metrix (PPM) [27], holds promise in this regard.

## Conclusions

This study has established that inter-observer agreement in pertrochanteric fracture classification, based on the Revised AO/OTA Classification, experiences a significant enhancement when employing 3D CT scan images compared to relying solely on plain radiographs. A plain radiographic evaluation displayed diminished consistency when contrasted with CT scanning, as it occasionally underestimated the grade of fracture classification and, on occasion, failed to detect lateral wall thickness and the presence of comminution fractures. The reliance on plain radiographs, if it leads to incorrect fracture type determination, may result in an erroneous implant selection.
